# Development of a hybrid artificial intelligence framework for pharmacotherapy optimization

**DOI:** 10.1007/s11096-026-02205-0

**Published:** 2026-08-21

**Authors:** Olaf Rose, Stephanie Clemens, Andreas Leiherer, Michael Bücker, Finn Petersson, Gerald Lirk, Christopher Mosch, Johanna Pachmayr, Kreshnik Hoti

**Affiliations:** 1 Institute of Pharmacy, Pharmaceutical Biology and Clinical Pharmacy, Paracelsus Medical University, Strubergasse 15, 5020 Salzburg, Austria; 2 Center of Public Health and Health Services Research, Paracelsus Medical University, Strubergasse 21, 5020 Salzburg, Austria; 3 Vorarlberg Institute for Vascular Investigation and Treatment (VIVIT), Carinagasse 47, 6800 Feldkirch, Austria; 4 Institute for Process Management and Digital Transformation (IPD), FH Münster, Corrensstraße 25, 48149 Münster, Germany; 5 University of Applied Sciences of Upper Austria, Softwarepark 11, 4232 Hagenberg, Austria; 6 Faculty of Medicine, Division of Pharmacy, University of Pristina, Str. Bulevardi I Dëshmorëve, 10000 Prishtina, Republic of Kosovo

**Keywords:** Artificial intelligence, Clinical pharmacy, Framework, Pharmacotherapy, Retrieval augmented generation

## Abstract

**Introduction:**

Pharmacotherapy optimization in multimorbid patients is increasingly complex due to polypharmacy, fragmented data, expanding electronic health records, and workforce constraints. Conventional clinical decision support systems remain largely rule-based and often fail to adequately incorporate patient-specific context. While artificial intelligence offers new opportunities, stand-alone models remain insufficiently reliable for high-risk pharmacotherapy decision support.

**Aim:**

To develop a relevance-driven, clinician-supervised hybrid AI framework for pharmacotherapy optimization.

**Method:**

Using a design science-informed approach, an interdisciplinary research group developed a conceptual framework for AI-supported pharmacotherapy optimization. Framework development was informed by prior feasibility work, published literature, clinical practice requirements, and iterative interdisciplinary discussions. Hybrid AI was defined as the combination of retrieval-augmented generation, deterministic safety rules, and large language model reasoning.

**Results:**

Seven design principles were identified, including decomposition of clinical activities, relevance-based prioritization, hybrid reasoning under clinician oversight, integration of patient goals, transparency of evidence sources, longitudinal optimization within a governed closed loop, and evaluation as a design requirement. These principles informed a conceptual architecture integrating structured clinical data, patient preferences, longitudinal patient information, and evidence retrieval within a clinician-governed decision-support framework.

**Conclusion:**

The proposed framework conceptualizes AI as a relevance-structuring, clinician-governed decision-support layer rather than an autonomous decision-maker. By combining hybrid reasoning, patient-specific context, and professional oversight, it provides a conceptual foundation for future development, implementation, and evaluation of AI-supported pharmacotherapy systems.

## Impact statements

- Conventional clinical decision support systems in pharmacotherapy remain largely rule-based and often fail to reflect patient-specific context, while stand-alone large language models present challenges regarding reliability and transparency.
- Seven design principles were identified for AI-supported pharmacotherapy optimization, resulting in a hybrid framework that combines retrieval-augmented generation (RAG), deterministic safety mechanisms, and contextual AI reasoning.
- The resulting framework incorporates a closed-loop architecture integrating longitudinal clinical data, patient-reported information, and patient preferences as explicit components of pharmacotherapy optimization.
- The framework conceptualizes AI as a clinician-governed, relevance-structuring decision-support layer, with responsibility for clinical decisions remaining with healthcare professionals.

## Introduction

Pharmacotherapy optimization in multimorbid patients is increasingly complex due to polypharmacy, expanding electronic health records (EHR), drug shortages, and workforce constraints, resulting in the need to make individualized decisions under time pressure [1–4]. Conventional clinical decision support systems (CDSS) remain largely rule-based and frequently generate alerts rather than context-sensitive reasoning, contributing to limited clinical impact and alert fatigue [5–11]. Pharmacotherapy optimization has evolved toward individualized, goal-oriented care that integrates patient preferences, clinical context, and interprofessional decision-making [12]. As complexity increases, approaches that structure and prioritize clinically relevant information may offer an alternative to traditional static rule-based decision support [13–15]. Artificial intelligence (AI) offers the potential to support this transition by enabling automated data integration and contextual interpretation. While specific models for pharmacotherapy optimization are scarce, AI is widely accepted among healthcare professionals [16–18]. The recently developed MedinAI guidelines highlight the need for transparent reporting of AI applications in medicines and pharmacotherapy, including model architecture, data sources, human oversight, and ethical considerations [19]. However, stand-alone models, particularly large language models (LLMs), remain unreliable in complex pharmacotherapy without structured clinical context and transparent justification [20–23]. Safe and clinically meaningful decision support therefore may require integration of AI with established safety mechanisms, longitudinal patient data, and clinician oversight.

### Aim

The aim of this study was to develop a relevance-driven, clinician-supervised hybrid AI framework for pharmacotherapy optimization. In this framework, hybrid AI combines retrieval-augmented generation (RAG), deterministic safety rules, and LLM-based reasoning. Specifically, the study sought to derive design principles and translate them into a conceptual architecture designed to support, rather than replace, clinical reasoning in pharmacotherapy optimization.

## Method

### Study design

**Fig. 1:**
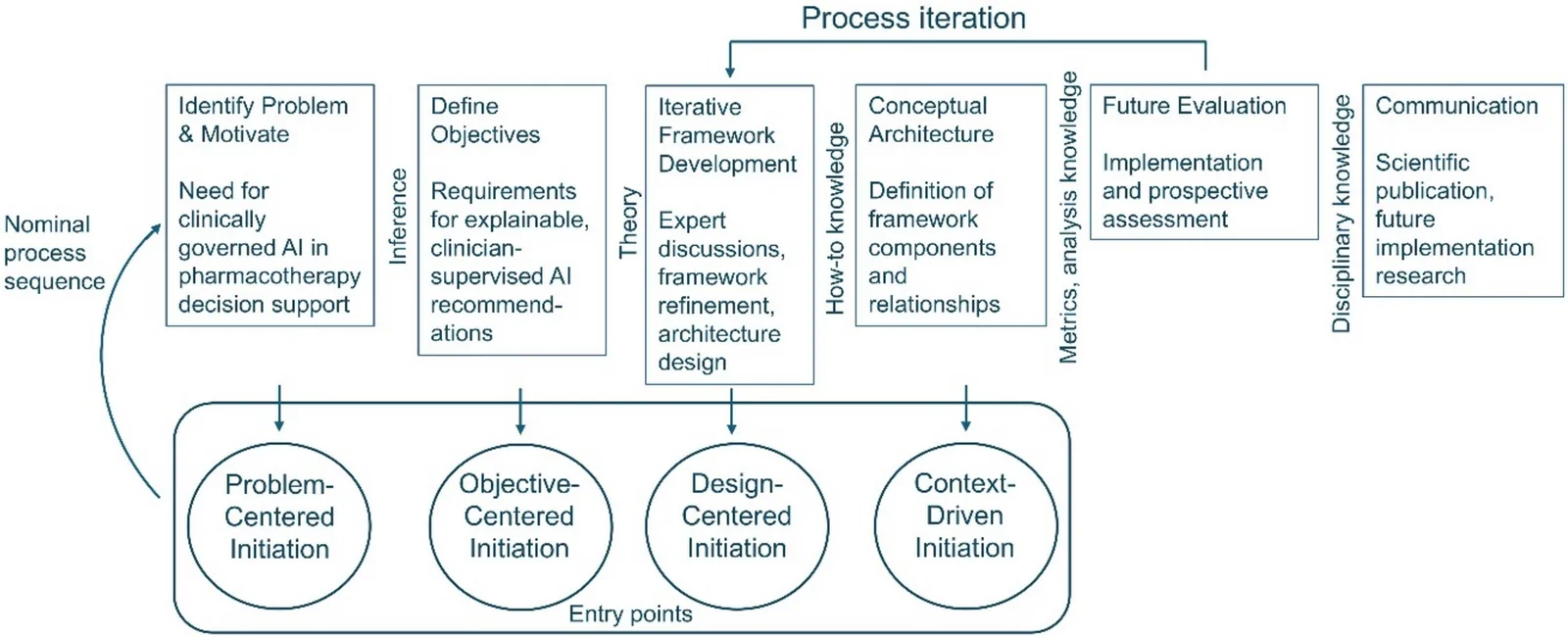
Application of selected design science research principles to framework development [24]

The proposed framework was guided by a set of design principles derived from early feasibility work, human factors research, and real-world constraints in clinical practice [20]. The framework development was informed by design science research (DSR) principles, as described by Peffers et al. and Gregor et al., particularly problem identification, artifact development, and iterative refinement, rather than following a fully predefined DSR process [24, 25]. Development was iterative and based on repeated discussions among members of the interdisciplinary research group. Iterations were not predefined, formally counted, or documented as separate consensus rounds. Accordingly, this work should be understood as a design science-informed conceptual framework rather than a formal consensus study. Progression between iterations was guided by emerging agreement regarding framework structure, clinical plausibility, and alignment with the identified design principles. Figure 1 depicts the application of DSR matrix on the framework development.

### Framework development process

The framework was developed between June 2025 and February 2026 by an interdisciplinary research group with expertise in clinical pharmacy, pharmacotherapy, artificial intelligence, digital health, and medication management. Development was informed by the group’s common ongoing research activities in creating an AI model for pharmacotherapy optimization, prior feasibility work in AI-assisted pharmacotherapy, literature on clinical decision support systems, and repeated discussions in individual meetings and group consultations. Initial framework elements were identified from recurring challenges in pharmacotherapy optimization, including relevance prioritization, integration of longitudinal patient data, incorporation of patient preferences, explainability of recommendations, and clinician oversight [26]. These elements were iteratively refined through discussion, visual modeling, and critical comparison with established requirements for safe and clinically meaningful decision support. The final framework represents the consensus position of the working group at the time of manuscript preparation.

### Ethics approval

According to institutional requirements at the Paracelsus Medical Private University, formal ethics approval was not required for this type of conceptual framework development.

## Results

### Design principles

The framework development process resulted in seven design principles for AI-supported pharmacotherapy optimization. These principles were derived from prior feasibility work, published literature on clinical decision support and AI in healthcare, recurring challenges in pharmacotherapy optimization, and iterative interdisciplinary discussions during framework development.

1. Decomposition of clinical activities.

Pharmacotherapy optimization consists of distinct clinical activities with different risk profiles, information requirements, and frequencies [12, 15, 26]. Decomposition may facilitate meaningful digital support and avoid indiscriminate automation of complex clinical decision-making [26]. For example, a comprehensive medication review can be decomposed into distinct tasks such as acquiring data, performing the patient assessment, setting therapeutic goals, assessing medication safety or evaluation of guideline adherence. Such decomposition enables targeted digital support while preserving clinician oversight of the overall decision-making process.

2. Relevance-based prioritization.

CDSS should prioritize information according to clinical relevance, patient vulnerability, and context-specific actionability. Within this framework, AI structures clinical relevance by identifying issues that most urgently require professional judgement rather than replacing decision-making. For example, a clinically significant drug-related problem associated with serious harm would be prioritized over minor informational alerts, allowing decision support to focus attention on issues most relevant to patient outcomes and therapeutic decision-making [20].

3. Hybrid reasoning under human oversight.

Safe pharmacotherapy decision support may benefit from hybrid reasoning that combines deterministic safety rules, established risk models, retrieval-augmented evidence, and contextual AI-based synthesis [20]. AI augments but does not replace clinical reasoning, and human oversight remains essential.

4. Explicit integration of patient goals and treatment burden.

Clinical relevance depends on patient priorities, including treatment goals, tolerability thresholds, symptom burden, and adherence barriers. Explicit integration of these factors is necessary to avoid technically correct but clinically misaligned recommendations.

5. Transparency and retained professional authority.

AI-generated recommendations must remain advisory, with clinicians retaining full authority to accept, modify, or reject them. Transparent presentation of underlying evidence supports verification, accountability, and informed decision-making [20].

6. Longitudinal pharmacotherapy optimization within a governed closed loop.

Pharmacotherapy optimization is dynamic and requires systems that capture outcomes, overrides, and safety events to enable iterative refinement. Continuous learning must occur within defined governance structures to ensure auditability, compliance, and trust.

7. Evaluation as a design requirement.

Evaluation should be embedded in system design and initially focus on usability, workflow integration, transparency, and reliability of recommendations. Only after stable use is demonstrated should evaluation extend to clinical outcomes and patient-level effects.

### Conceptual framework

This framework is intended to inform governance, design, and evaluation of AI-supported pharmacotherapy rather than to prescribe a specific technical implementation. The conceptual framework described below operationalizes the design principles outlined above by translating them into an integrated architecture for AI-supported pharmacotherapy optimization. It positions hybrid AI as an augmentation layer in pharmacotherapy, supporting clinicians in managing therapeutic complexity, reducing cognitive and administrative burden, and enabling focus on patient interaction. To this end, the framework proposes the integration of structured clinical data, guideline-based evidence, and patient-specific preferences to support medication-related clinical decision-making.

### Information foundation and multimodal inputs

Pharmacotherapy optimization requires a longitudinal and integrative clinical perspective. Relevant information extends beyond the current medication list and may, depending on availability, include diagnoses, laboratory values, renal and hepatic function, vital signs, pharmacogenomics, comorbidities, and prior treatment courses. Increasing availability of EHRs enables access to many of these data points, while patient preferences and symptom trajectories provide complementary insight into lived treatment experience. Clinical guidelines and safety recommendations frequently convey therapeutic logic through flow charts, tables, and visual algorithms. Consequently, the proposed framework is based on a multimodal information foundation that integrates structured clinical data, unstructured clinical narratives via natural language processing, patient-reported information, and guideline artefacts via computer vision-based representation learning. By aligning heterogeneous information sources within a unified representation, the system aims to support context-aware reasoning while minimizing additional documentation burden for clinicians.

### Dual engine “hybrid” reasoning: combination with knowledge base

**Fig. 2:**
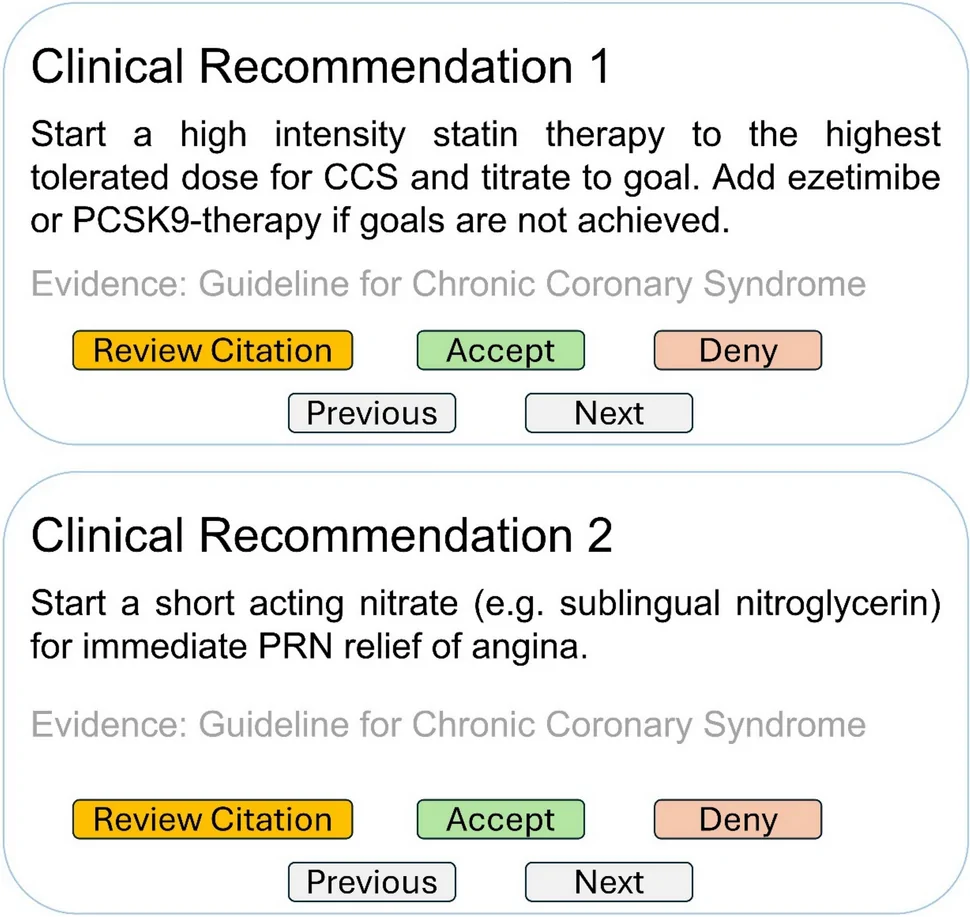
Example of a user interface with dual engine reasoning, providing the source of the recommendation. CCS: chronic coronary syndrome, PCSK9: proprotein convertase subtilisin/kexin type 9, PRN: pro re nata/as needed

Safe and clinically meaningful recommendations require explicit grounding in established therapeutic evidence. RAG enables LLM to reference up—to-date guideline content, product information, safety communications, and curated evidence repositories at the point of care [27, 28]. Within the proposed framework, hybrid reasoning combines contextual AI-based synthesis with deterministic safety rules and established risk models. Together, these components are intended to support transparent and evidence-anchored recommendations (Fig. 2). Uncertainty signaling is integral to the system design. When data are incomplete or evidence is limited, the system should explicitly communicate uncertainty and prompt clinical verification. Hybrid reasoning is intended to augment, not replace, professional judgment. To support reliable and traceable evidence retrieval, source documents such as clinical guidelines require systematic preparation. Large documents are converted into machine-readable formats, stripped of non-informative elements, and segmented into semantically coherent units (chunking). These units are indexed individually to enable precise contextual retrieval.

### Integration of patient goals, patient preferences and treatment burden

Modern pharmacotherapy requires alignment with patient goals, values, tolerability and daily capabilities. Adherence is influenced by symptom severity, regimen complexity, health literacy and expectations. Therefore, the framework integrates structured documentation of patient goals and burden thresholds, such as preferred treatment intensity, acceptable side effect levels, priorities related to cognition, mobility or sleep and willingness to engage in monitoring. Incorporating these parameters ensures personalized alignment rather than algorithm-centric optimization. For CDSS, this important information on complaints, preferences and treatment goals needs to be translated into relevant and contextualized prompts. A digital scale is required to prepare these inputs for the AI system. However, patient-reported inputs may be incomplete or imprecise, and data collected by wearables or other devices are often unspecific and not routinely integrated into medication reviews. An application on a mobile device, for example an avatar-based digital assistant, could elicit medication-related preferences, collect longitudinal data, and complement information obtained during patient-clinician encounters. This digital health assistant may also provide guidance when predefined red-flag patterns are detected.

### Human oversight, autonomy and accountability

Professional responsibility for medication decisions remains with the clinician. AI within the proposed framework serves an advisory function and does not operate autonomously. Pharmacists, physicians and other health professionals retain full authority to accept, modify, or reject system-generated recommendations based on clinical judgment, patient context, and interprofessional considerations. To support accountable oversight, recommendations are accompanied by transparent reasoning, linked evidence sources, and concise rationale summaries. Explicit uncertainty signaling informs clinicians when a system-generated recommendation should not be applied directly without further review. In such cases, the signal prompts clinicians to reassess the underlying data, consider missing patient information, or seek additional evidence before making a final decision. These mechanisms are intended to preserve professional agency while enhancing efficiency and situational awareness. By embedding decision authority, explanation, and documentation within existing workflows, the framework aligns digital support with ethical obligations, regulatory expectations, and established standards of professional practice [19]. Moreover, the framework enables clinicians to provide feedback on system recommendations, which can be used to evaluate the model’s performance, facilitate learning, and continuously validate and refine the AI, ensuring its ongoing improvement.

### The clinician governed closed loop AI model

**Fig. 3:**
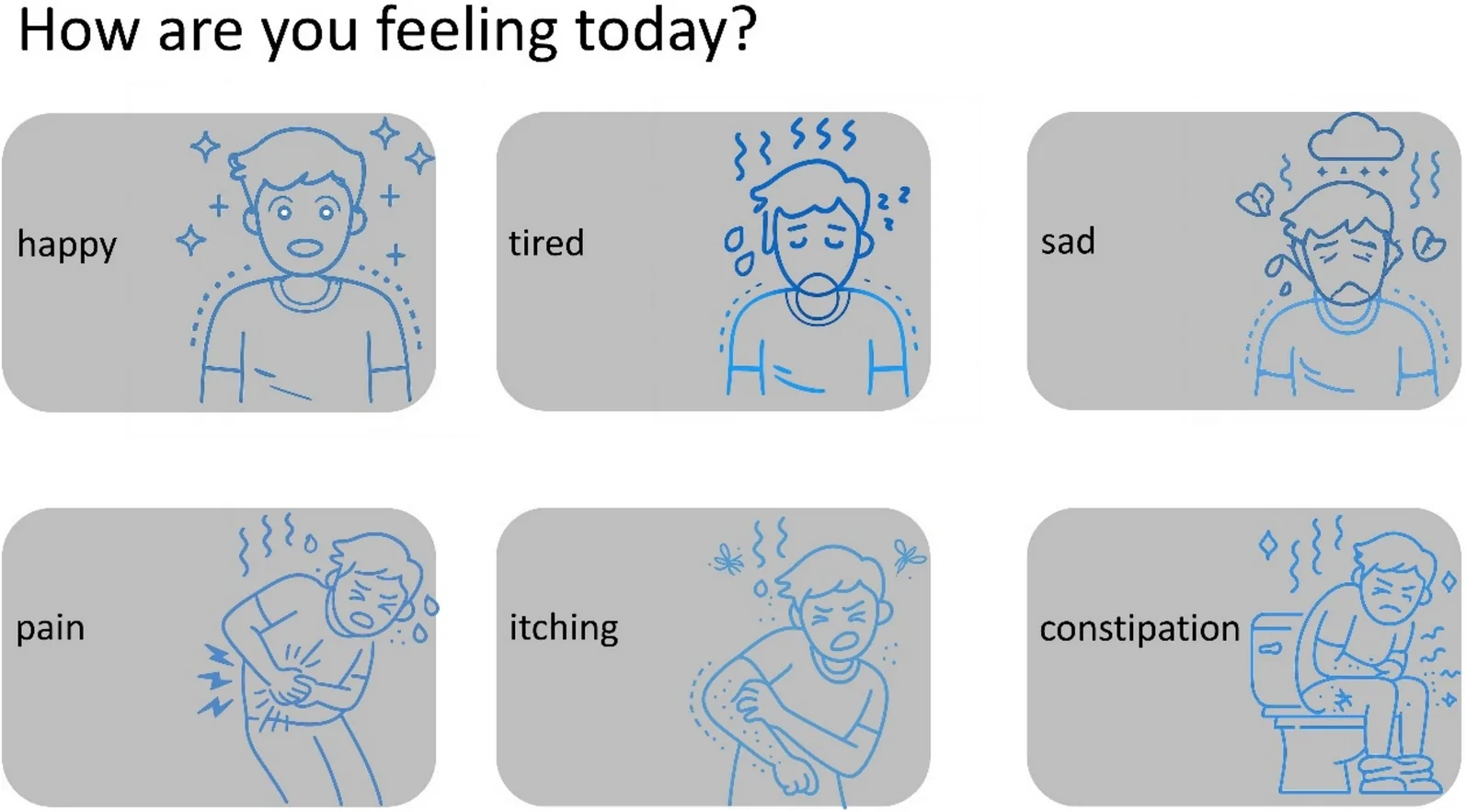
Example of a digital patient feedback panel, which can be augmented with a talking avatar

Pharmacotherapy optimization is inherently dynamic. Patient conditions evolve, therapies change, and new information emerges over time. The proposed framework therefore suggests to adopt a closed-loop architecture that supports longitudinal monitoring and iterative refinement of recommendations. As illustrated in Fig. 3, patient-reported information on symptoms, goals, well-being, medication-related problems, and adherence could be captured longitudinally through digital interfaces.

These inputs complement data obtained during clinical encounters and enable early identification of emerging issues that may otherwise remain unrecognized between visits. Beyond a standard patient-facing interface, an avatar-based assistant may facilitate a more structured and continuous capture of patient-reported information. It can add information for the categorization of chief complaints and treatment-related concerns, translate them into clinically relevant representations, and enhance patient understanding of therapy and medication use. By providing tailored guidance and timely feedback, it may contribute to improved adherence. In addition, the system can generate alerts and prompt referrals to clinicians when predefined red-flag patterns are detected. Information retrieved through the interface is fed back into the AI model, thereby closing the loop for pharmacotherapy optimization.

**Fig. 4:**
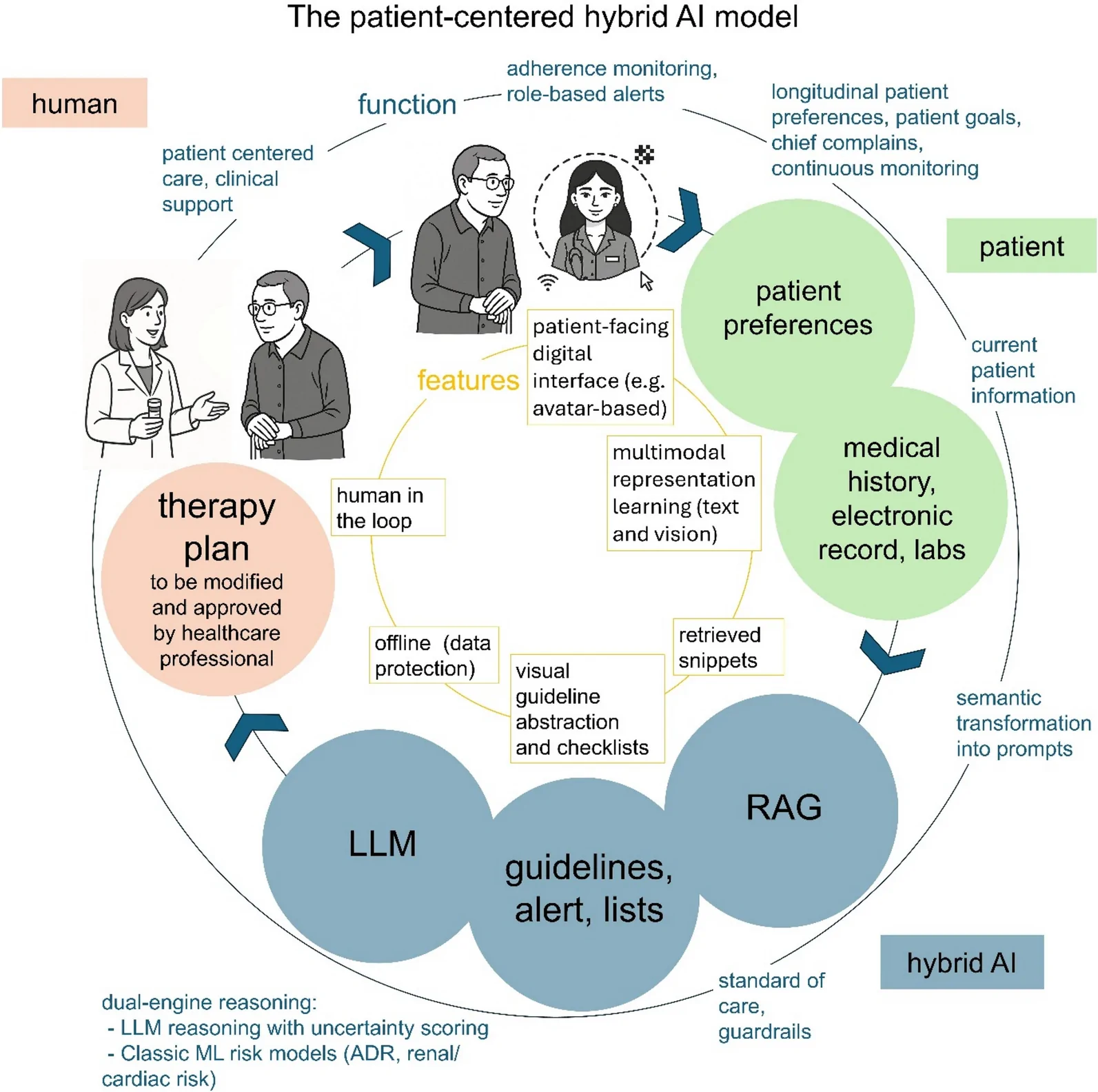
Patient-centered hybrid AI model designed as a clinician-governed closed loop. ADR: adverse drug reaction, AI: artificial intelligence, LLM: large language model, ML: machine learning, RAG: retrieval augmented generation

Multimodal clinical inputs, including structured electronic health record data, laboratory results, medication plans, patient preference measures, and guideline artefacts, would be continuously integrated into the system. Evidence layers based on RAG access to clinical guidelines, summaries of product characteristics, and safety communications inform a hybrid reasoning process that combines contextual AI-based synthesis with classical risk models and uncertainty scoring. A conceptual operationalization of the proposed hybrid AI architecture, including the closed-loop mechanism, is shown in Fig. 4.

System-generated therapy recommendations remain subject to human oversight. Digital support can facilitate adherence reminders and track symptoms. Patients may actively signal concerns or alerts to the care team through the same interface. Clinician actions, patient feedback, and observed outcomes are documented to support transparency, accountability, and iterative system refinement. Within defined governance structures, this feedback enables continuous learning on priorities, while ensuring that adaptation remains evidence-grounded, auditable, and aligned with professional standards rather than uncontrolled self-optimization.

### Safety, audit and continuous learning

Robust safety mechanisms are a foundational requirement for digital decision support in pharmacotherapy optimization. Within the proposed framework, safety guardrails include verification of doses, contraindications, and clinically relevant drug-drug interactions, as well as mechanisms to limit hallucinations and unsupported inferences in AI-assisted reasoning, thereby providing the required level of reliability and helping to uphold ethical standards in clinical decision making [19]. All clinician interactions with system-generated recommendations, including overrides, modifications, errors, and adverse events, should be systematically documented. This audit trail may enable traceability, accountability, and structured safety monitoring across real-world use. Feedback loops can support iterative improvement based on observed performance while preserving transparency and professional responsibility. Continuous learning can be constrained by governance structures. System adaptation ideally remains evidence-grounded and does not rely on unconstrained data generation or autonomous self-optimization. Deployment within privacy-preserving, jurisdiction-compliant infrastructures and centrally validated, version-controlled updates should ensure regulatory alignment, auditability, and prevention of uncontrolled model drift. By combining safety verification, auditability, and supervised AI, the framework aims to increase transparency, strengthen clinician trust, and support safe longitudinal use in routine clinical practice.

## Discussion

This conceptual framework proposes a shift toward AI as a supervised decision-support layer for pharmacotherapy optimization, rather than as a substitute for professional judgment. The framework builds on three central propositions:

First, pharmacotherapy optimization in multimorbid patients is unlikely to be adequately captured by rule execution or single-model inference. It requires longitudinal context, evolving evidence, explicit patient goals, and clinical reasoning about trade-offs under uncertainty. Second, expanding digital infrastructure, including EHR access and external digital biomarkers, can improve access to relevant information but also increases cognitive load. Effective AI support should reduce routine cognitive work and preserve clinicians’ time for patient counselling and shared decision-making. Third, safe implementation requires transparent, clinician-supervised systems. Stand-alone LLMs remain unreliable in complex pharmacotherapy, while they can offer excellent answers to single questions and support prioritization of relevance. These considerations are consistent with emerging guidance for the development and evaluation of AI systems in healthcare. Recent reporting and evaluation frameworks, including CONSORT-AI, TRIPOD-AI, and the recently published MedinAI guidance, emphasize transparency, human oversight, explainability, and accountability as central requirements for trustworthy clinical AI systems [19, 29, 30]. Amugongo et al. however found a lack of standardized evaluation frameworks for RAG-based applications [31], while several previous studies have largely focused on evaluating the outputs of existing consumer LLMs [23]. In contrast, this framework for hybrid, RAG-based AI was developed from experiences with a de novo hybrid RAG-based system specifically designed for pharmacotherapy optimization, which incorporates several safeguards that are not typically met by LLMs, nor by stand-alone AI systems [20]. Hybrid architectures that combine evidence retrieval, safety rules, and contextual AI reasoning through RAG may offer a more robust and accountable approach [28].

However, hybrid architecture does not eliminate important limitations of AI systems [27, 31]. Recent studies have highlighted challenges related to retrieval quality, hallucinations, explainability, uncertainty communication, and dependency on underlying evidence sources. These limitations reinforce the need for transparent evidence retrieval, clinician oversight, and prospective evaluation in real-world pharmacotherapy settings.

The central contribution of this framework is to conceptualize AI in pharmacotherapy not as an autonomous decision-maker, but as a clinician-governed reasoning infrastructure that prioritizes relevance, transparency, and patient-defined goals. It can guide further development of AI models for pharmacotherapy optimization. Future evaluation should include multiple outcome layers, including system reliability, alert relevance, clinician acceptance, workflow impact, and downstream clinical outcomes such as drug-related problems, adverse drug events, and treatment alignment with patient goals.

### Implications

Pharmacotherapy optimization differs fundamentally from many other areas of clinical decision support and AI employment. Rather than pattern recognition, it requires context-dependent reasoning about competing risks, treatment goals, and patient preferences. As digital infrastructures increasingly shape clinical decision-making, the question is not only whether AI will be used in pharmacotherapy, but who defines the clinical logic that governs it. If clinicians involved in pharmacotherapy optimization do not actively participate in the design, validation, and governance of such systems, therapeutic priorities may increasingly be influenced by algorithmic processes that are not fully aligned with pharmacotherapeutic reasoning, patient priorities, or professional standards [32–34]. Ensuring that AI-supported decision support reflects pharmacotherapeutic reasoning and patient-centered care is therefore a core responsibility of the clinical community.

### Limitations

This paper is conceptual and does not provide validation of the proposed framework or model. It synthesizes feasibility insights, practice-based constraints, and emerging evidence, but does not report implementation outcomes or performance metrics. The design principles reflect the consensus of the interdisciplinary working group, but the iterative discussions were not predefined, formally documented, or independently validated; this limits reproducibility and should be addressed in future structured consensus and empirical validation studies. The framework should therefore be interpreted as an architectural blueprint. The proposed evaluation dimensions are intended to guide implementation and research rather than substitute for formal clinical studies. Future research should assess usability, workflow integration, patient experience, safety performance, and override behavior across diverse care settings. Key implementation challenges include interoperability with EHR, secure data processing, explainability standards, and alignment with medical device regulation. Implementation is expected to be modular, beginning with deterministic safety rules before integrating more advanced AI components. Although RAG methods and uncertainty estimation strategies exist, their performance in multimorbid pharmacotherapy contexts remains to be demonstrated. Ethical issues, including bias monitoring, transparency, and equitable access, also warrant systematic examination. Although patient goals, treatment burden, and patient-reported outcomes are central components of the proposed framework, patients were not directly involved in the development, review, or appraisal of the framework. Future work should include patient and public involvement to evaluate whether the proposed architecture adequately reflects patient priorities, preferences, and expectations regarding AI-supported pharmacotherapy.

## Conclusion

This study resulted in a relevance-driven, clinician-supervised hybrid AI framework for pharmacotherapy optimization that integrates hybrid reasoning, patient-specific context, and clinician governance. Therapeutic complexity, expanding electronic health records, workforce strain, and rising expectations for clinical contribution have challenged traditional approaches to pharmacotherapy optimization. Manual optimization may become increasingly difficult to scale in response to growing therapeutic complexity, expanding clinical data, and workforce constraints. This framework outlines a conceptual blueprint for a clinician-supervised, multimodal hybrid AI framework embedded in a digital closed loop with the patient, designed to augment, rather than replace, professional judgement. A central feature of the framework is the integration of patient-specific clinical data, longitudinal information, and patient preferences to support individualized pharmacotherapy optimization. The proposed framework provides a conceptual foundation to guide future research, implementation, and evaluation of AI-supported pharmacotherapy optimization systems.

## Data Availability

The dataset of this study is available on rational request from the corresponding author.
